# Occurrence and Anastomosis Grouping of *Rhizoctonia* spp. Inducing Black Scurf and Greyish-White Felt-Like Mycelium on Carrot in Sweden

**DOI:** 10.3390/jof7050396

**Published:** 2021-05-19

**Authors:** Shirley Marcou, Mariann Wikström, Sara Ragnarsson, Lars Persson, Monica Höfte

**Affiliations:** 1 Department of Plants and Crops, Faculty of Bioscience Engineering, Ghent University, 9000 Ghent, Belgium; Shirley.Marcou@UGent.be; 2 Agro Plantarum AB, Kärrarpsvägen 410, S-265 90 Åstorp, Sweden; mariann.wikstrom@agroplantarum.se; 3 Swedish Board of Agriculture, Elevenborgsvägen 4, S-234 56 Alnarp, Sweden; sara.ragnarsson@jordbruksverket.se; 4 Brandsberga Gård AB/Agri Science Sweden AB, Brandsberga Gård 210, S-264 53 Ljungbyhed, Sweden; lars@agriscience.se

**Keywords:** *Rhizoctonia* spp., Sweden, AG 3, *Daucus carota* L. (carrot), *Solanum tuberosum* L. (potato), black scurf, greyish-white felt-like mycelium

## Abstract

Carrots with different *Rhizoctonia*-like symptoms were found in the main Swedish carrot production areas from 2001–2020. The most commonly observed symptoms were a greyish-white felt-like mycelium and black scurf, the latter often associated with *Rhizoctonia solani* anastomosis group (AG) 3-PT on potato. An overall increase in disease incidence in all studied fields over time was observed for both symptoms. The majority of *Rhizoctonia* isolates sampled from carrot in the period 2015–2020 were identified as AG 3 (45%) and AG 5 (24%), followed by AG 1-IB (13%), AG 11 (5%), AG-E (5%), AG BI (3%), AG-K (3%) and AG 4-HGII (2%). To our knowledge, this is the first report describing AG 5 in Sweden as well as AG 3, AG 11 and AG-E inducing *Rhizoctonia*-like symptoms on carrot. Secondly, we report for the first time that *R. solani* AG 3, and the less observed AGs: AG 1-IB and AG 5 can induce black scurf symptoms on the taproot of carrots. Due to a widely used carrot-potato crop rotation in Swedish areas, a possible cross-over from potato to carrot is suggested. This information is of high importance to reduce *Rhizoctonia* inoculum in soils, since avoiding carrot-potato crop rotations needs to be considered.

## 1. Introduction

Domesticated carrot (*Daucus carota* L. subsp. *sativus* (Hoffm.) Schubl. & G. Martens) is a biennial plant belonging to the *Apiaceae* family [1]. It is cultivated worldwide for the fresh market and processing industry for its nutritive taproot. In Sweden, carrot and potato are the most cultivated root and tuber crops. In 2019, carrots and potatoes were produced on approximately 1700 ha (109,000 tons) and 23,650 ha (846,900 tons), respectively, in Sweden. The main Swedish production areas of carrots are Scania (57%) and Gotland (25%). Potatoes are mainly grown in the southern provinces of Scania, Halland and Blekinge (62%) [2]. Cultivated carrots are mainly grown in open fields and are mostly sown in Swedish areas from March to mid-June. Carrots for direct consumption and cold storage are harvested between early July and late October [3]. There are also carrots stored under straw that are harvested during the winter from December to May. A wide range of disease symptoms have been described worldwide during carrot cultivation, including root and stem rot, seedling damping-off, leaf spot and blight [4]. Damping-off and root rot can induce serious disease problems on carrot in Sweden and are caused by a species complex of three genera: *Pythium* species (spp.), *Fusarium* spp. and *Rhizoctonia* spp. [5].

The genus *Rhizoctonia* is a highly complex and heterogeneous group of basidiomycete fungi, which does not produce any asexual conidia [4]. *Rhizoctonia* spp. are found in nature primarily as vegetative mycelium and sclerotia. Only occasionally, under specific environmental conditions, sexual basidiospores are formed [6]. *Rhizoctonia* isolates are currently classified into three major groups based on differences in the number of nuclei per hyphal cell: uninucleate *Rhizoctonia* (teleomorph: *Ceratobasidium*), binucleate *Rhizoctonia* (teleomorph: *Ceratobasidium* and *Tulasnella*) and multinucleate *Rhizoctonia* (teleomorph: *Thanetophorus* and *Waitea*) [7,8]. Each major group is taxonomically further divided into different anastomosis groups (AGs) based on the fusion of touching hyphae. Moreover, these AGs can be subdivided in subgroups on the basis of high similarity in pathogenicity, genetic characteristics or the frequency of fusion between isolates [6,7,8,9].

From all groups, multinucleate *Rhizoctonia solani* Kühn (teleomorph: *Thanatephorus cucumeris*) is the most extensively studied and widely recognized species. This highly destructive soil-borne fungus can attack several distinct plant parts such as seedlings, roots, tubers, stems, leaves and fruits [4,10]. Commonly observed symptoms are pre- and post-emergence damping-off, root and stem rot, stem canker and black scurf [9]. To date, 14 AGs have been defined within multinucleate *R. solani* species: AG 1 to AG 13 and AG BI [6,7,11,12,13]. On the other hand, binucleate *Rhizoctonia* spp. are currently grouped into the following AGs: AG-A to AG-S [6], AG-T and AG-U [14]. However, AG-T and AG-U were confirmed by Sharon et al. [7] to belong to AG-A and AG-P, respectively. Additionally, AG-J and AG-N are excluded from *Rhizoctonia* [7], and representative isolates of AG-M are lost [6]. Two new binucleate groups have recently been reported in China and were defined as AG-V [15] and AG-W [16]. Therefore, of the 23 binucleate *Rhizoctonia* AGs described, only 18 AGs are currently known. To date, the most accurate method for classification of multinucleate and binucleate *Rhizoctonia* isolates into AGs and subgroups is ribosomal DNA (rDNA)-internal transcribed spacer (ITS) sequence analysis [7].

On carrot, different AGs and subgroups are distinguished as causal agents of diseases but *R. solani* AG 2–2 and AG 4 are the most frequently observed, in which AG 2–2 is often associated with root rot and AG 4 with seedling damping-off [17]. In Europe, AG 1(-IB), AG 2–1, AG 2–2(-IIIB), AG 4(-HGII) and AG 6 have been described and are associated with lesions on the taproot and on seedlings and with seedling damping-off [17,18,19]. In the rest of the world, more specifically, in the United States, New Zealand and Japan, AG 1(-IB/-IC), AG 2–1, AG 2–2(-IV), AG 2–4, AG 4(-HGI/-HGII), AG 5, AG 9 and AG BI have been described [20,21,22,23,24,25,26,27,28,29,30]. Binucleate *Rhizoctonia* isolates of AG-C, AG-D and AG-K induced root rot symptoms on carrot in New Zealand [30] but caused only minor damage. AG-U induced black scurf symptoms on carrot in Japan [24].

In Sweden, carrot is often cultivated in a narrow crop rotation system with potato (*Solanum tuberosum* L.). On potato, different AGs of multinucleate and binucleate *Rhizoctonia* spp. have been described. However, *R. solani* AG 3-PT is considered as the main cause of disease in potato and has been described in different regions to be inducing black scurf and stem canker symptoms [31,32]. However, AG 2–1, AG 4-HGII, AG 5, AG 8, AG-A to AG-E, AG-K and AG-R can also inflict substantial damage and black scurf on potato [31,32,33]. Stem and stolon canker have been found among others in association with AG 2–2-IIIB, AG 3, AG 5, AG 4-HGI/-HGIII, AG-A and AG-R [33,34]. In Sweden, both AG 3 and AG 2 are found on potato, but AG 2 is considered less aggressive, due to the fact that AG 2 forms fewer sclerotia on the tubers than other AGs [35,36].

Considering the increasing importance of Rhizoctonia diseases in carrot and potato in Sweden, there is a need for more knowledge to understand the distribution and spread of this fungal pathogen. During a field survey of over 20 years, the occurrence of *Rhizoctonia*-like symptoms was investigated in different Swedish carrot fields in the main growing areas. During the last six years, *Rhizoctonia* isolates were collected from diseased carrot plants to identify the AGs and subgroups infecting these carrots, using rDNA-ITS sequence analysis. Throughout the field survey, over the last six years, similar *Rhizoctonia*-like symptoms were noticed on potatoes grown in the same fields as the carrots and in potato fields in other regions. *Rhizoctonia* was also isolated from these crops to compare the anastomosis groups from potato with those from carrot. In addition, the pathogenic potential of a selection of isolates was tested toward carrot and potato.

## 2. Materials and Methods

### 2.1. Field Surveys, Sample Collection and Rhizoctonia Isolation

Over the period 2001–2020, between 12 and 49 carrot fields in Sweden were investigated from August to September of each year for the occurrence of Rhizoctonia diseases. The investigated fields are situated in Scania, Halland and Gotland, the main growing areas of Southern, Western and Eastern Sweden, respectively. A total number of 489 fields were considered for sampling. The occurrence of different *Rhizoctonia*-like symptoms was observed for each field. During the last six years, from 2015 to 2020, selected carrot parts with *Rhizoctonia*-like symptoms were plated on Potato Dextrose Agar (PDA; Difco, BD Diagnostics, Stockholm, Sweden), amended with the antibiotic streptomycin sulphate (100 mg/L), to isolate *Rhizoctonia* for further identification. From 2018 to 2020, disease symptoms caused by *Rhizoctonia* spp. on black nightshade (*Solanum nigrum* L.) and some other weeds in carrot fields were noted as well. Infected plant tissues were placed on PDA amended with streptomycin sulphate for isolation of *R. solani*. In addition, potato plants with *Rhizoctonia*-like symptoms were investigated during the last three years, and *Rhizoctonia* was isolated from infected plant tissues in the same way. The investigated potato plants have grown either in the same fields as the carrots or in potato fields in Southern Sweden, Gotland, Västergötland or Dalarna.

### 2.2. Determination of Anastomosis Groups: Phylogenetic Analysis

All of the 55 *Rhizoctonia* isolates (Table 1) obtained from the three different crops or plants were grown on liquid Potato Dextrose Broth (PDB; Difco, BD Diagnostics, Erembodegem, Belgium) at room temperature. After 5 days, the mycelium was recovered from the medium by filtration and ground in liquid nitrogen using a Retsch MM 400 mixer mill (Retsch GmbH, Haan, Germany) and steel beads. Genomic DNA was extracted using a commercially available DNA-extraction kit (Invisorb^®^ Spin Plant Mini Kit; Stratec, Berlin, Germany) and subsequently stored at −20 °C. The quality and concentration of the DNA solution was determined with a spectrophotometer (DS-11, DeNovix, Wilmington, DE, USA). The primer pairs ITS4 (5′-TCCTCCGCTTATTGATATGC-3′) and ITS5 (5′-GGAAGTAAAAGTCGTAACAAGG-3′) were used for the amplification of the nuclear rDNA-ITS fragment, including the 5.8S rDNA gene [37]. Polymerase chain reactions (PCR) were performed with a total reaction volume of 50 μL per sample, using a Flexcycler PCR Thermal cycler (Analytik Jena GmbH, Jena, Germany). *Taq* polymerase (Promega, Madison, WI, USA) was used supplemented with 10 µL of Colorless GoTaq^®^ Reaction buffer (5x, Promega), 1 µL 10 mM dNTP mix, 27.7 µL nuclease free water, 3.5 µL 10 µM of each ITS primer and 40 ng of target DNA. Amplification was conducted by an initial denaturation step at 94 °C for 10 min, followed by 35 cycles at 94 °C for 1 min, 55 °C for 1 min and 72 °C for 1 min. Cycling ended with a final extension step at 72 °C for 10 min. Amplification products were separated by electrophoresis (100 V, 30 min) on a 1% agarose gel in a 0.5X Tris-acetate-EDTA (TAE) buffer and subsequently visualised by ethidium bromide staining on a UV transilluminator. Before sequencing, enzymatic clean-up of the amplified PCR products took place with ExoSAP-ITTM (Thermo Fisher Scientific, Waltam, MA, USA) in accordance with manufacturer instructions. All amplicons obtained from the *Rhizoctonia* isolates were sent to LGC Genomics GmbH (Berlin, Germany) and sequences of both strands were determined using Sanger sequencing. Consensus sequences of all *Rhizoctonia* isolates were created using BioEdit v. 7. To determine the AGs of the isolates, the obtained rDNA-ITS consensus sequences were compared with the database of representative sequences from Sharon et al. [7], using the BLASTn tool. Sequences from this gene region were deposited in GenBank as accession numbers MW999148–MW999205. Alignments for the *Rhizoctonia* isolates were constructed using MUSCLE, implemented in MEGA 8 [38]. The phylogenetic tree was built using the same representative isolate sequences from Sharon et al. [7].

### 2.3. Morphological Characteristics

The morphology of all 55 *Rhizoctonia* isolates (Table 1) was investigated on Potato Dextrose Agar (PDA; BD Diagnostics, Erembodegem, Belgium). A mycelium plug of a 7-day-old colony, grown at room temperature (19–22 °C) was transferred to the middle of a new PDA plate. Incubation took place at room temperature (19–22 °C), and pictures were taken after 14 days.

### 2.4. Pathogenicity Tests

A subset of nine *R. solani* isolates and one binucleate *Rhizoctonia* isolate was tested for their pathogenicity towards carrots in two greenhouse tests. One isolate of AG 1-IB (RhCaGo-34), six isolates of AG 3 (RhCaES-19, RhPoWS-32, RhCaES-60, RhCaES-61, RhCaES-75 and RhBnES-80), one isolate of AG 5 (RhCaES-62), one isolate of AG BI (RhCaGo-39) and one binucleate *Rhizoctonia* isolate of AG-E (RhCaWS-46) were chosen for these tests. The isolates were inoculated on wheat kernels according to the method described by Scholten et al. [39]. Briefly, water-soaked wheat kernels were autoclaved for 1 h on two successive days and then inoculated with three fungal discs (Ø 6 mm), cut at the edge of a recently grown *Rhizoctonia* colony cultured on PDA. Flasks containing the inoculated kernels were incubated for 21 days at room temperature and shaken every 2–3 days to avoid coagulation. Kernels used for inoculation had equivalent sizes. A mixture of sand and peat soil (P-jord, Hasselfors Garden AB, Örebro, Sweden) was used in the trials in a 50:50 ratio. Five inoculated wheat kernels were placed in the middle of each pot. A control treatment was set up with non-inoculated, sterile wheat kernels. Ten carrot seeds (cultivar Romance F1, Nunhems) were sown in each pot. Before sowing, the carrot seeds were surface-sterilized in a sodium hypochlorite solution consisting of 1 part common laundry bleach (2.7% sodium hypochlorite) to 2 parts clear water, for 2 min, and were then thoroughly rinsed in water. In both tests, four pots per isolate were used. The pots were placed in a greenhouse at 20 °C, with 16 h lighting. After three weeks, the number of germinated plants were counted, and the plant heights were measured. After about 10 weeks, all roots were washed and a disease severity index (DSI), ranging from 0 (completely white, healthy roots) to 100 (dead plants), was assessed on each root [40]. In the second greenhouse test, three additional isolates were included as positive control isolates (Table 2), since *R. solani* AG 2–1 and AG 4-HGII are described in literature as causal agents of seedling damping-off [17,25,27]. From all symptomatic plants, plant tissues were placed on PDA for re-isolation of *R. solani.*

Subsequently, another subset of six different *R. solani* isolates (RhCaES-19, RhCaES-22, RhCaES-38, RhPoWS-30, RhPoWS-32 and RhPoHa-49), all belonging to AG 3 except for isolate RhCaES-38 (AG 5), was tested for in vitro and in vivo pathogenicity towards carrots. Two assays were set up, in which *R. solani* isolate BK004–1-1 (AG 4-HGII) was used as a positive control on carrot [17]. In both assays, carrot seeds (cultivar Nantes) were surface-sterilized before use in 1% sodium hypochlorite solution during 30 s and rinsed three times with sterile demineralised water. In the in vitro assay (trial 1), six surface-sterilized seeds were germinated on Gamborg B5 medium (including vitamins, Duchefa) in a square Petri dish. Two mycelial disks (Ø 6 mm) from recently grown *R. solani* cultures on PDA were placed between the seeds. Disks of sterile PDA medium were used as a control treatment. One half of the Petri dish was each time covered with aluminium foil to protect the roots from light. The dishes were placed in an upright position in a growth chamber (18 °C; 16 h light), and incubation for seed germination initially took place in the dark for two days. The number of germinated seedlings was recorded and the disease severity was assessed at 10 days post inoculation (dpi), using the following scale: score 0 = no damage; score 1 = minor discoloration on stem, hypocotyl, root or leaf; score 2 = discoloration and small necrotic lesions (<1 mm Ø) on stem, hypocotyl, root or leaf; score 3 = discoloration and large necrotic lesions (≥1 mm Ø) on stem, hypocotyl, root or leaf; score 4 = seedling dead. Disease severity index was calculated ranging from 0 to 100, as follows: [(0 × number of seedlings within class 0) + (1 × number of seedlings within class 1) + (2 × number of seedlings within class 2) + (3 × number of seedlings within class 3) + (4 × number of seedlings within class 4)] × 100/(total numbers of plants within treatment × 4). A completely randomized design was applied with three Petri dishes (six seedlings each) per *R. solani* isolate. In the in vivo assay (trial 2), twenty surface-sterilized seeds of carrot cultivar Nantes were sown in two rows in a perforated plastic box (22 × 15 × 6 cm) filled with sand and potting soil (universal type 1 Structural, Snebbout N.V., Belgium) in a 50:50 ratio. These seedlings were inoculated with 10 *Rhizoctonia*-colonised wheat kernels in the middle of the box [39]. Control seedlings were similarly treated with sterile wheat kernels. The seedlings were incubated at 18 °C, with a photoperiod of 16 h light and 8 h dark. Two repetitions were used per treatment. The number of germinated seedlings and the plant length were respectively counted and measured at 22 dpi.

Finally, the same subset of six *R. solani* isolates (RhCaES-19, RhCaES-22, RhCaES-38, RhPoWS-30, RhPoWS-32, RhCaES-38 and RhPoHa-49) were chosen to test their pathogenicity towards potato. A potato tuber (cultivar Bintje) was put in the middle of a plastic pot filled with sand and potting soil (universal type 1 Structural, Snebbout N.V., Belgium) in a 50:50 ratio. Four wheat kernels were placed around the potato tuber. The wheat kernels used for inoculation were produced according to the above-mentioned method, described by Scholten et al. [39]. A control treatment was set up with non-inoculated, sterile wheat kernels. The potato tubers were put in a growth chamber (18 °C; 16 h light) to observe the germination of the sprouts and were harvested 33 days post inoculation. Damage of the sprouts was numerically categorized as follows: score 0 = no damage, no lesions; score 1 = minor damage, one to several lesions (< 5 mm); score 2 = intermediate damage, lesions (>5 mm) and girdling of some sprouts; score 3 = major damage, large lesions, girdling and death of most sprouts; and score 4 = all sprouts killed. The formation of sclerotia on the potato tubers was also investigated after carefully washing the potato tubers with tap water. Six repetitions were used per treatment.

### 2.5. Data Analysis

All statistical tests were performed at a confidence level of *p* = 0.05. The trend lines in disease incidence in carrot fields were analyzed by linear regression. The data of the greenhouse tests performed on carrots were statistically analyzed using the SAS/Stat (Statistical Analyses System) one-way analysis and the Duncan multiple range test to determinate the significance between the treatments. The data of the other pathogenicity tests were analyzed with non-parametric Kruskal Wallis and Mann–Whitney comparisons, using the software package SPSS 25.0.

## 3. Results

### 3.1. Field Survey, Sample Collection, Morphological Characteristics and Identification of Rhizoctonia Isolates

**Figure 1:**
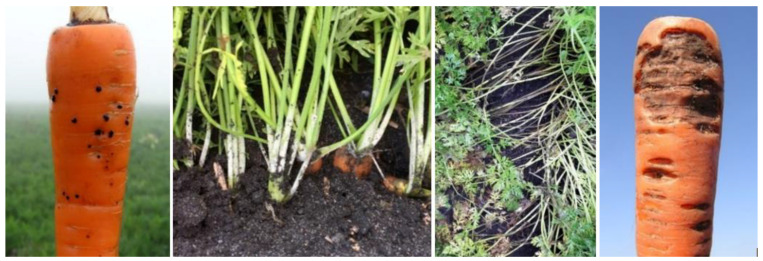
Different types of *Rhizoctonia*-like symptoms observed in the studied carrot fields, from left to right: black scurf, greyish-white felt-like mycelium, brown wilted stem bases/leaves and crown rot.

To investigate the occurrence of Rhizoctonia diseases in the main carrot-growing areas of Sweden (Western and Eastern Scania, Halland and Gotland), a total number of 489 fields were surveyed since 2001. Four types of symptoms were observed in many different fields: black scurf, greyish-white felt-like mycelium, crown rot and brown wilted stem bases/leaves (Figure 1). While brown wilted stem bases and/or leaves were found in many different fields, among which most often *R. solani* was isolated, and crown rot was only occasionally found. Next to *Rhizoctonia* spp., also *Fusarium* spp. and other fungi were isolated from these kinds of symptoms. However, the two most observed symptoms in the fields were black scurf and greyish-white felt-like mycelium, and *R. solani* was consistently isolated from carrots showing these symptoms. In 2020, these two types of symptoms were found in 50% of the investigated fields.

**Figure 2:**
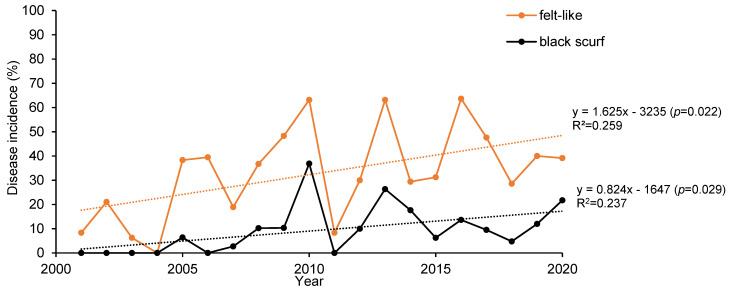
Disease incidence of *Rhizoctonia* symptoms in a total of 489 investigated carrot fields in Sweden during 2001–2020. Symptoms are divided in felt-like fungal growth and black scurf.

The disease incidence over time for *Rhizoctonia* black scurf symptoms and greyish-white felt-like fungal growth symptoms in Swedish carrot fields is shown in Figure 2. The trendlines indicate a significant increase in incidences of both types of *Rhizoctonia* symptoms from 2001 to 2020. The average disease incidence of black scurf increased from 0 to 17% of the investigated carrot fields (*p* = 0.029), and the average disease incidence of felt-like fungal growth increased from 18 to 49% of the fields (*p* = 0.022).

**Figure 3:**
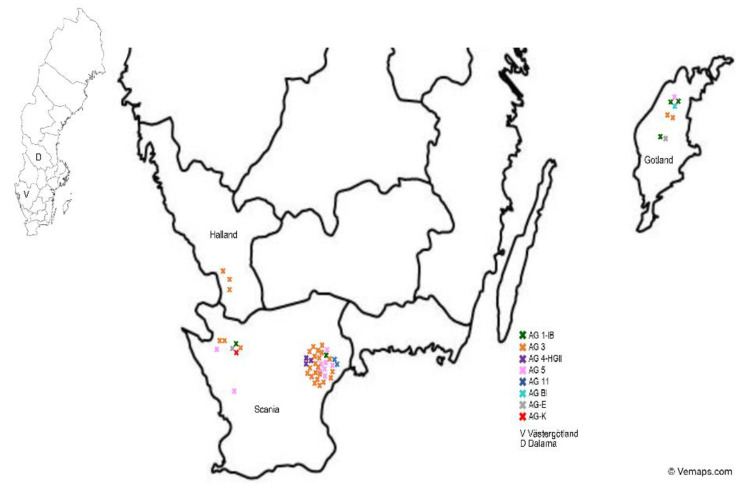
Map of Sweden showing the isolation sites and the different AGs of the *Rhizoctonia* isolates obtained from carrots, potatoes and black nightshade in different areas (V = Västergötland, D = Dalarna, Gotland, Halland and Scania).

Within the period 2015–2020, fifty-five samples were taken from diseased carrot, potato and black nightshade plants for further characterization. The investigated potato tubers and/or plants were grown either in the same fields as the carrots or in potato fields in Southern Sweden, Gotland, Västergötland or Dalarna. During the performed field surveys, black nightshade plants with *Rhizoctonia*-like symptoms were observed on the carrot fields, and some samples were also kept for further identification. A map of Sweden showing the isolation sites and the different AGs found on these three crops is visualized in Figure 3. 

**Figure 4:**
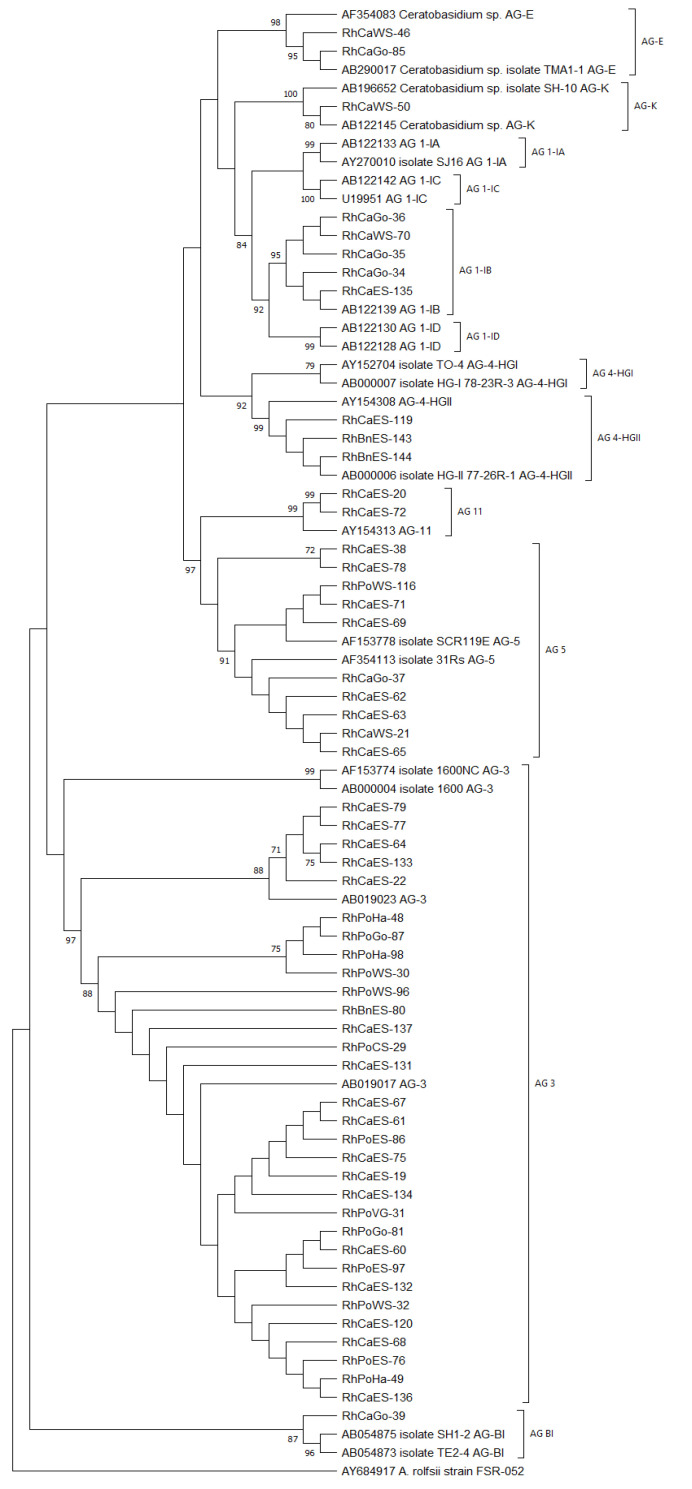
rDNA-ITS phylogeny of 52 multinucleate *Rhizoctonia solani* spp. and 3 binucleate *Ceratobasidium* spp. sampled from potato, carrot and black nightshade grown in different regions of Sweden. The maximum likelihood tree is derived from the alignment of these 55 *Rhizoctonia* isolates, 24 reference isolates from Sharon et al. [7] and the outgroup *Athelia rolfsii* (AY684917). Isolates with the Rh prefix were obtained from Sweden. Bootstraps are only given for those branches with bootstrap support higher than 70.

Anastomosis grouping of the 55 *Rhizoctonia* isolates was done using the sequence of the ITS-5.8S rDNA region (Table 1). A maximum likelihood phylogenetic tree with bootstrap 1000 was constructed derived from the alignment of 24 representative isolates from Sharon et al. [7] and our 55 isolates (Figure 4). 

Of the 55 *Rhizoctonia* isolates, three isolates were binucleate (two nuclei per hyphal cell) and belonged to AG-E (2/3) and AG-K (1/3) with a sequence similarity of 98–99% and 99%, respectively, compared with the representative isolates. A total of 52 isolates were multinucleate *R. solani* species, of which 5 isolates corresponded to AG 1-IB, 31 isolates to AG 3, 3 isolates to AG 4-HGII, 10 isolates to AG 5, 2 isolates to AG 11 and 1 isolate to AG BI. The sequence similarities within each multinucleate AG were as follows: AG 1-IB: 99–100%, AG 3: 99–100%, AG 5: 94–100%, AG 11: 94–95% and AG BI: 96%.

**Figure 5:**
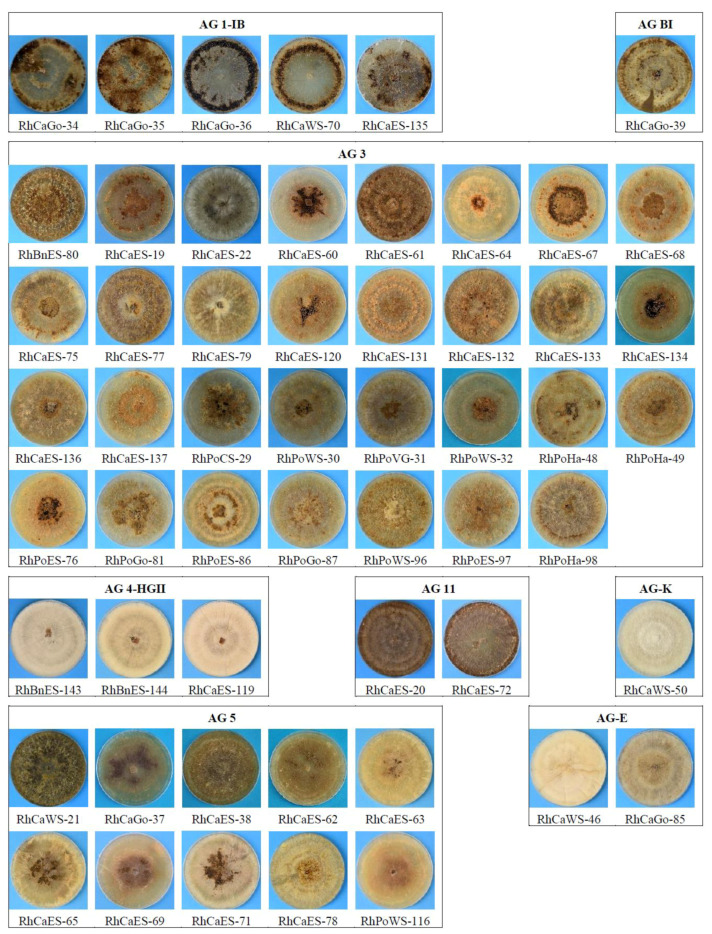
Colony morphology on PDA of different anastomosis groups: AG 1-IB, AG 3, AG 4-HGII, AG 5, AG 11, AG BI, AG-E and AG-K. Mycelium was 14 days old and incubated at room temperature.

The growth of the different *Rhizoctonia* isolates on PDA revealed differences in morphology for isolates belonging to the same or different AGs (Figure 5). The mycelium of isolates belonging to AG 4-HGII and AG-K was paler in comparison with the other studied AGs. Sclerotia formation was not observed for both AGs after an incubation period of 14 days. A longer incubation period was needed for sclerotia to develop (data not shown). While all other AGs produced sclerotia, isolates belonging to AG 3 and AG 5 were producing the sclerotia more in the center of the Petri dish, close to the inoculation source. On the other hand, isolates of AG 1-IB produced a more darkly pigmented mycelium, and sclerotia formation took place at the edge of the plate or completely spread over the plate. No other differences in mycelium morphology and sclerotia formation for isolates belonging to different AGs could be observed.

**Figure 6:**
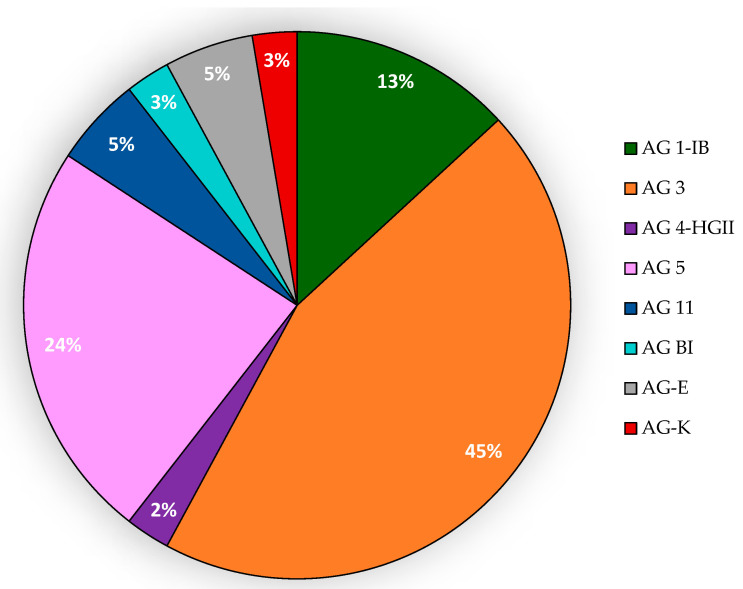
The distribution of AGs among the isolates sampled from carrot in the different Swedish areas.

Of the three isolates sampled from black nightshade, two of them belong to AG 4-HGII and one to AG 3. The distribution of AGs among the isolates sampled from carrot is depicted in Figure 6. In total, 69% of the isolates were identified as AG 3 or AG 5, of which AG 3 is the predominant group (65%). All potato isolates belong to AG 3, except for isolate RhPoWS-116, which was identified as AG 5. The majority of the isolates for these studied crops corresponded to *R. solani* AG 3.

**Figure 7:**
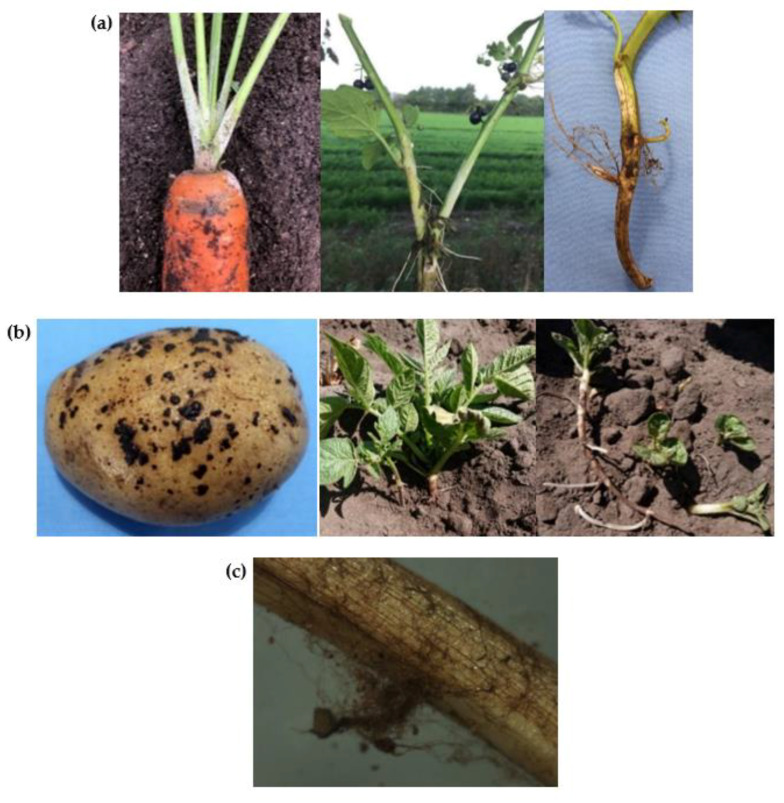
Different symptoms caused by *Rhizoctonia solani* AG 3. (**a**) Greyish-white felt-like mycelium symptoms on carrot (left), black nightshade (center) and potato (right); (**b**) Black scurf (left) and stem canker (center, right) on potato tubers and stolons; (**c**) Brown net of mycelium on carrot leaf stems.

During field observations in six Swedish areas, different *Rhizoctonia*-like symptoms on carrot, potato and black nightshade could be associated with *R. solani* AG 3 (Figure 7). Black scurf symptoms on the tap root were observed on carrots infected with AG 1-IB, AG 3 and AG 5, while the same symptoms on potato tubers were only caused by AG 3. Greyish-white felt-like mycelium was commonly observed on the three crops, mainly induced by AG 3. On carrots, also AG 5 and AG 11 could induce these felt-like mycelium symptoms. Brown wilting of the leaves and/or stem bases was noticed on carrots infected with AG 1-IB, AG 3, AG 4-HGII, AG 5, AG BI and AG-E. These symptoms were not observed on potato or black nightshade. Stem canker was observed on potatoes infected with AG 3 and AG 5, of which the latter showed typical rust-colored stolons. Less commonly observed symptoms were a brownish net of mycelium on a carrot plant infected with AG 3 and crown rot on carrot induced by AG-E and AG-K and on potato by AG 3.

### 3.2. Pathogenicity Assays

The pathogenicity towards carrot for a subset of isolates was tested, as can be seen in Table 3 and Table 4. The most aggressive isolates towards carrot seedlings were RhSbSS-17 and BK004-1-1 (both AG 4-HGII), inducing pre- and post-emergence damping-off. Isolate RhCfWS-83 (AG 2–1) induced browning of the roots. In the first two greenhouse assays (Table 3), none of the tested isolates reduced the emergence or plant height in early developmental stages of the carrots. Only isolate RhCaGo-34 (AG 1-IB) caused a slightly higher, but significant, DSI in comparison with the untreated control. Black scurf symptoms (black sclerotia) were found on carrots inoculated with RhCaES-19 and RhBnES-80 (both AG 3). Re-isolations of *Rhizoctonia* from carrots in the greenhouse tests were successfully performed with all AG 3 and AG 5 isolates. Only two of the isolates, RhCaGo-34 (AG 1-IB) and RhCaGo-39 (AG BI), could not be re-isolated from carrots. Re-isolates from carrots inoculated with RhCaES-60 and RhCaES-62 were identified again as AG 3 and AG 5, respectively, fulfilling Koch’s postulates. 

In the additional pathogenicity tests (Table 4), the DSI of all tested *R. solani* isolates were significantly different from the DSI of the aggressive control treatment (BK004-1-1 belonging to AG 4-HGII), which means that all tested *R. solani* AG 3 and AG 5 isolates were less pathogenic towards carrot seedlings. All tested isolates only caused minor discoloration (DSI between 9.4 and 18.3) and no seedling damping-off was observed, resulting in a rather weak pathogenic potential.

**Figure 8:**
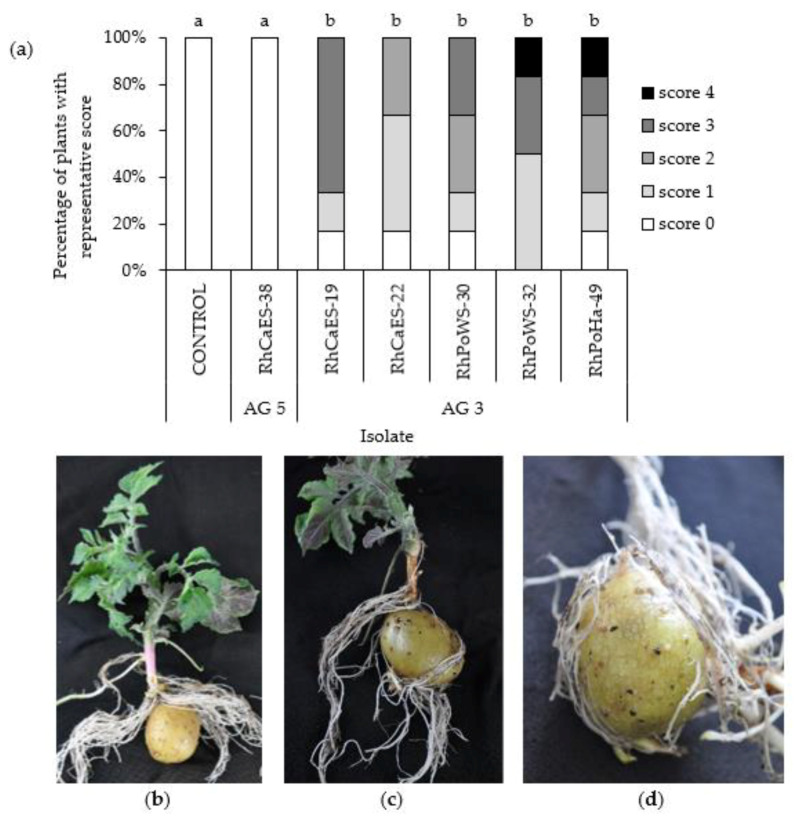
Pathogenicity towards potato (cv. Bintje) of six *R. solani* isolates belonging to AG 3 and AG 5, isolated from carrot and potato. (**a**) Percentage of plants with *Rhizoctonia* symptoms, 33 dpi (*n* = 6). Plants were scored using a scale ranging from 0 (no damage) to 4 (all sprouts dead). Data were statistically analyzed using Mann–Whitney tests. The different letters above the bars indicate significant differences in pathogenicity between isolates (*p* = 0.05); (**b**) a healthy plant: no symptoms induced by isolate RhCaES-38 (AG 5); (**c**) black scurf symptoms on potato tubers and girdling of the stem base induced by isolate RhPoWS-32; (**d**) formation of black scurf on potato tubers by isolate RhCaES-19 (AG 3).

Secondly, a small subset of six isolates belonging to AG 3 or AG 5 and isolated from carrot and potato was tested to verify if they were pathogenic towards potato. The disease evaluation at 33 dpi for the different *R. solani* isolates is shown in Figure 8a. Anastomosis group 5 isolate RhCaES-38 did not induce any symptoms and can be considered as non-pathogenic towards potato (Figure 8b), while lesions (often > 5 mm) and girdling on/of potato sprouts were seen for all the other AG 3 isolates (Figure 8c), including the isolates obtained from carrot (RhCaES-19 and RhCaES-22). Formation of black sclerotia on potato tubers was observed for all AG 3 isolates (Figure 8d). Black scurf was not induced on the tuber by AG 5 (RhCaES-38).

## 4. Discussion

To understand the distribution and occurrence of Rhizoctonia diseases in the main carrot production areas in Sweden, field surveys were conducted during the last 20 years. Two major *Rhizoctonia*-like symptoms were found: black scurf and a greyish-white felt-like mycelium. The results indicate that the disease occurrence of *Rhizoctonia solani* inducing these symptoms have increased over the period of time (Figure 2). Possibly, part of the increasing trendline can be explained by the increased production of carrots in Eastern Scania. In 2001, the acreage for carrot cultivation was about 100 hectares, while, in 2020, the acreage was about 700 hectares [41]. Another part of the increase in *Rhizoctonia*-like symptoms can be explained by changes in seed treatment and foliar sprays. The active substance iprodione, with well-studied effect against *R. solani* in many different crops, has been one of the standard fungicides for carrot seed treatment and for tuber treatment in potatoes, for many years [42,43,44,45,46]. Tuber treatment with iprodione has been banned in Sweden since 2010, but the seed treatment in carrots was available until 2018 through imported carrot seeds [47,48]. Since iprodione was withdrawn, metalaxyl-M and thiram have been the standard seed treatment in carrots. However, these active substances are not effective against *R. solani* [4]. In potato, iprodione was replaced by other active substances with good effect against *R. solani* [49]. Iprodione was also used as a foliar spray in carrots and other crops in the plant rotation with carrots and potatoes until 2010, but the dose, the number of applications and the crops in which it was registered were decreased in 2003 [48]. The reduced use in 2003 and the loss of iprodione in 2010 and 2018 may play a role in the increase of *Rhizoctonia*-like symptoms.

The dips in disease incidence for both black surf and greyish-white felt-like mycelium in certain years (Figure 2) might be explained by unusual weather conditions. In December 2010, the average temperature in the investigated area in Sweden was 8 °C below the normal temperature, which was −6.2 °C for 2010, in comparison with the average temperature of +1.7 °C for 2001–2020. In the summer of 2018, the temperature was unusually high, and there was an extreme drought. Despite these dips, the increase in the occurrence of *R. solani* during the 20 years is significant. The fact that the symptoms were found in 50% of the total investigated fields during the last year means that Rhizoctonia diseases are seriously progressing in Swedish areas. The accurate characterization and identification of isolates that cause *Rhizoctonia*-like symptoms in carrot and potato is required and will be more important in the future to understand the dynamics of the pathogen.

In the current study, most of the isolates obtained from black scurf and greyish-white felt-like mycelium symptoms were identified as AG 3-PT. Greyish-white felt-like mycelium is probably the sexual stage of AG 3, as often observed in potato [31,50]. Potatoes with black scurf symptoms and stem canker were also sampled in our study and were often associated with *R. solani* anastomosis group AG 3-PT, which is also confirmed by other studies [31,32,33,50]. All tested *R. solani* isolates that belong to AG 3 were pathogenic toward potato and weakly pathogenic toward carrot, suggesting a cross-over from potato and carrot and vice versa. In the field, clear black scurf symptoms were observed on both crops mainly induced by *R. solani* AG 3. Together with the observation that carrot is an alternative host and can help *R. solani* AG 3 to survive in the absence of potato [51,52], a crop rotational strategy in carrot-potato is not recommended. A further observation is that, in these growing areas in Sweden, the soils are mainly sandy (Table 1), and *R. solani* spreads better in sandy soils, compared with other soil types [53]. In addition to AG 3, binucleate AG-E and AG-K and multinucleate AG 1-IB, AG 3, AG 4-HGII, AG 5, AG 11 and AG BI were found in our study. However, from all the Swedish isolates collected from carrot, potato and black nightshade, the majority belonged to *R. solani* AG 3-PT (56%), followed by AG 5 (18%). The growth of the different isolates on PDA resulted in differences in morphology for isolates belonging to the same or different AGs, except for AG 4-HGII, which can be distinguished by a paler mycelium colour in comparison with the other studied AGs. To date, sequencing the ITS-5.8S rDNA region seems to be the most appropriate method for *Rhizoctonia* spp. classification [7,54].

As indicated before, almost all isolates sampled from potato belong to AG 3-PT (except for one isolate belonging to AG 5). The distribution of AG 3 isolates sampled from potato was not restricted to a particular region in Sweden, indicating a dispersed occurrence of *R. solani* AG 3 in the different studied regions. *Rhizoctonia solani* other than AG 3, including AG 5, have been reported in association with potato in different parts of the world [30,32,33,55,56]; however, to our knowledge, AG 5 has not been reported in Sweden yet [5]. In Swedish areas, both AG 3 and AG 2 have already been identified on potato [36,57], but future investigations are necessary to study the occurrence and importance of *R. solani* AG 5 in Swedish areas.

In carrot, similar to potato, AG 3-PT was the most predominant group found in Sweden. Next to AG 3, AG 1-IB and AG 5 could also cause black scurf on the taproot of carrots. Black scurf has been noted in Japan, caused by binucleate AG-U [24]. Until now (2021), no reports were found in literature on *R. solani* AG 1-IB, AG 3 and AG 5 inducing black scurf in carrot crops. Often, the wilting of the leaves and/or brown wilted stem carrot bases were observed in fields in Gotland, Western and Eastern Scania, caused by the previous mentioned AGs, including AG BI and AG-E. Recently, the same symptoms referred as leaf blight and petiole rot were found in Japan, induced by AG 1-IB [58]. In addition, AG 5 has often been related to canker lesions in the US [25] and AG BI to root rot in New-Zealand [30], but these AGs have never been found to induce wilting symptoms. Subsequently, as far as known, AG 3 and AG-E have not been described before to induce disease symptoms on carrot.

Finally, our study also confirms the ability of black nightshade to harbour *R. solani* as reported by Jager et al. [59]. This suggests that probably a wide range of *R. solani* AGs can be harboured by weeds such as black nightshade, meaning that weed control is important in controlling *Rhizoctonia* inoculum in the soil or in the fields.

To our knowledge, carrot black scurf caused by *R. solani* AG 3, AG 5 and AG 1-IB has not been previously reported from any other carrot-producing country in Europe, nor outside Europe. Secondly, this is the first report describing AG 3, AG 11 and AG-E inducing *Rhizoctonia*-like symptoms on carrots. The wilting of stems or leaves and the occurrence of greyish-white felt-mycelium symptoms on the stems did not affect the edible taproot of the carrots, indicating that these symptoms may not result in large yield losses. However, as discussed by Mori et al. [58], due to disease damage on the leaves, mechanically harvesting can indirectly result in huge economic losses by impeding the harvesting of the carrot crops. Our largest concern is the impact of the carrot crop as an alternative host of AG 3 in crop rotation with potato, which can have a negative impact on this crop. The information from this study is of high importance in order to reduce *Rhizoctonia* inoculum in the soil, since avoiding carrot-potato crop rotation systems needs to be considered.

## Figures and Tables

**Table 1:** Origin and identification of *Rhizoctonia* isolates obtained from black nightshade (Bn), carrot (Ca) and potato (Po) in different regions of Sweden ^1^.

Isolate	Host Plant	Year of Isolation	Region	Soil Type	Symptoms	Anastomosis Group	Accession Number
RhBnES-80	Black nightshade	2018	Eastern Scania	Sand	Greyish-white felt-like mycelium	AG 3	MW999148
RhBnES-143	Black nightshade	2020	Eastern Scania	Loam	Rust coloured stem	AG 4-HGII	MW999149
RhBnES-144	Black nightshade	2020	Eastern Scania	Loam	Rust coloured stem	AG 4-HGII	MW999150
RhCaGo-34	Carrot	2017	Gotland	Calcium mud	Brown wilted stem bases/leaves	AG 1-IB	MW999151
RhCaGo-35	Carrot	2017	Gotland	Calcium mud	Brown wilted stem bases/leaves	AG 1-IB	MW999152
RhCaGo-36	Carrot	2017	Gotland	Calcium mud	Brown wilted stem bases/leaves	AG 1-IB	MW999153
RhCaWS-70	Carrot	2018	Western Scania	Loamy sand	Brown wilted stem bases/leaves	AG 1-IB	MW999154
RhCaES-135	Carrot	2020	Eastern Scania	Loamy sand	Black scurf	AG 1-IB	MW999155
RhCaES-19	Carrot	2015	Eastern Scania	Loamy sand	Greyish-white felt-like mycelium	AG 3	MW999156
RhCaES-22	Carrot	2015	Eastern Scania	Loamy sand	Greyish-white felt-like mycelium	AG 3	MW999157
RhCaES-60	Carrot	2018	Eastern Scania	Sand	Greyish-white felt-like mycelium	AG 3	MW999158
RhCaES-61	Carrot	2018	Eastern Scania	Sand	Brown net of mycelium on leaf stems	AG 3	MW999159
RhCaES-64	Carrot	2018	Eastern Scania	Sand	Greyish-white felt-like mycelium	AG 3	MW999160
RhCaES-67	Carrot	2018	Eastern Scania	Sand	Brown wilted stem bases/leaves	AG 3	MW999161
RhCaES-68	Carrot	2018	Eastern Scania	Sand	Greyish-white felt-like mycelium	AG 3	MW999162
RhCaES-75	Carrot	2018	Eastern Scania	Sand	Black scurf	AG 3	MW999163
RhCaES-77	Carrot	2018	Eastern Scania	Sand	Greyish-white felt-like mycelium	AG 3	MW999164
RhCaES-79	Carrot	2018	Eastern Scania	Sand	Greyish-white felt-like mycelium	AG 3	MW999165
RhCaES-120	Carrot	2020	Eastern Scania	Loamy sand	Brown wilted stem bases/leaves	AG 3	MW999166
RhCaES-131	Carrot	2020	Eastern Scania	Sand	Greyish-white felt-like mycelium	AG 3	MW999167
RhCaES-132	Carrot	2020	Eastern Scania	Sand	Brown wilted stem bases/leaves	AG 3	MW999168
RhCaES-133	Carrot	2020	Eastern Scania	Loam	Brown wilted stem bases/leaves	AG 3	MW999169
RhCaES-134	Carrot	2020	Eastern Scania	Sand	Black scurf	AG 3	MW999170
RhCaES-136	Carrot	2020	Eastern Scania	Sand	Black scurf	AG 3	MW999171
RhCaES-137	Carrot	2020	Eastern Scania	Loamy sand	Black scurf	AG 3	MW999172
RhCaES-119	Carrot	2019	Eastern Scania	Sand	Brown wilted stem bases/leaves	AG 4-HGII	MW999173
RhCaWS-21	Carrot	2015	Western Scania	Loam	Greyish-white felt-like mycelium	AG 5	MW999174
RhCaGo-37	Carrot	2017	Gotland	Peat	Brown wilted stem bases/leaves	AG 5	MW999175
RhCaES-38	Carrot	2017	Eastern Scania	Sand	Black scurf	AG 5	MW999176
RhCaES-62	Carrot	2018	Eastern Scania	Sand	Brown wilted stem bases/leaves	AG 5	MW999177
RhCaES-63	Carrot	2018	Eastern Scania	Sand	Brown wilted stem bases/leaves	AG 5	MW999178
RhCaES-65	Carrot	2018	Eastern Scania	Sand	Brown wilted stem bases/leaves	AG 5	MW999179
RhCaES-69	Carrot	2018	Eastern Scania	Sand	Greyish-white felt-like mycelium	AG 5	MW999180
RhCaES-71	Carrot	2018	Eastern Scania	Sand	Brown wilted stem bases/leaves	AG 5	MW999181
RhCaES-78	Carrot	2018	Eastern Scania	Loamy sand	Brown wilted stem bases/leaves	AG 5	MW999182
RhCaES-20	Carrot	2015	Eastern Scania	Sand	Greyish-white felt-like mycelium	AG 11	MW999183
RhCaES-72	Carrot	2018	Eastern Scania	Sand	Greyish-white felt-like mycelium	AG 11	MW999184
RhCaGo-39	Carrot	2017	Gotland	Calcium mud	Brown wilted stem bases/leaves	AG BI	MW999185
RhCaWS-46	Carrot	2017	Western Scania	Loamy sand	Crown rot	AG-E	MW999186
RhCaGo-85	Carrot	2018	Gotland	Calcium mud	Brown wilted stem bases/leaves	AG-E	MW999187
RhCaWS-50	Carrot	2016	Western Scania	Loamy sand	Crown rot	AG-K	MW999188
RhPoCS-29	Potato	2017	Dalarna	Moraine	Black scurf	AG 3	MW999189
RhPoWS-30	Potato	2017	Western Scania	Loamy sand	Black scurf	AG 3	MW999190
RhPoVG-31	Potato	2017	Väster-götland	Loamy sand	Black scurf	AG 3	MW999191
RhPoWS-32	Potato	2017	Western Scania	Loamy sand	Black scurf	AG 3	MW999192
RhPoHa-48	Potato	2017	Halland	Sand	Black scurf	AG 3	MW999193
RhPoHa-49	Potato	2017	Halland	Sand	Black scurf	AG 3	MW999194
RhPoES-76	Potato	2018	Eastern Scania	Sand	Greyish-white felt-like mycelium	AG 3	MW999195
RhPoGo-81	Potato	2018	Gotland	Sand	Black scurf	AG 3	MW999196
RhPoES-86	Potato	2018	Eastern Scania	Sand	Black scurf	AG 3	MW999197
RhPoGo-87	Potato	2018	Gotland	Sand	Black scurf	AG 3	MW999198
RhPoWS-96	Potato	2020	Western Scania	Loamy sand	Stem canker	AG 3	MW999199
RhPoES-97	Potato	2020	Eastern Scania	Sand	Stem canker	AG 3	MW999200
RhPoHa-98	Potato	2020	Halland	Sand	Stem canker	AG 3	MW999201
RhPoWS-116	Potato	2020	Western Scania	Sand	Rust coloured stolons	AG 5	MW999202

^1^ ES = Eastern Scania; Go = Gotland; WS = Western Scania; CS = Central Sweden; VG = Västergötland; Ha = Halland.

**Table 2:** Positive control strains sampled from different hosts ^1^ in different Swedish regions ^2^.

Isolate	Host Plant	Year of Isolation	Region	Identity (%)	Query Cover (%)	Reference Accession nb. [7]	Anastomosis Group	Accession Number
RhSbSS-17	Sugarbeet	2016	Southern Scania	100	100	AB000006	AG 4-HGII	MW999203
RhOsSS-66	Oil rapeseed	2018.	Southern Scania	97	100	AB054850	AG 2–1	MW999204
RhCfWS-83	Cauliflower	2018	Western Scania	99	99	AB054850	AG 2–1	MW999205

^1^ Sb = sugarbeet; Os = oil rapeseed; Cf = cauliflower; ^2^ WS = Western Scania; SS = Southern Sweden.

**Table 3:** Pathogenicity of ten *Rhizoctonia* isolates towards young carrot plants (cv. Romance F1), performed in two greenhouse trials. In the second trial, RhOsSS-66 and RhCfWS-83 (both AG 2–1) and RhSbSS-17 (AG 4-HGII) were used as positive control treatments.

Trial No.	Anastomosis Group	Isolate No.	Symptom on Original Host Plant	Emerged Plants (%) ^1,2^	Plant Height (cm) ^2^	Disease Severity Index (0–100) ^2^	Black Scurf
1	Untreated	-	-	84 n.s.	10.8 abc	8.8 bc	-
	AG 1-IB	RhCaGo-34	brown wilted stem bases/leaves	80 n.s.	11.3 ab	11.3 a	-
	AG 3	RhCaES-19	greyish-white felt-like mycelium	90 n.s.	11.6 ab	8.5 bc	observed
	AG 3	RhCaES-60	greyish-white felt-like mycelium	98 n.s.	10.7 bc	7.2 bc	-
	AG 3	RhCaES-61	brown net of mycelium on leaf stems	88 n.s.	11.1 abc	6.9 c	-
	AG 3	RhCaES-75	black scurf	93 n.s.	11.0 abc	8.8 bc	-
	AG 3	RhBnES-80	greyish-white felt-like mycelium	88 n.s.	12.0 a	9.8 ab	observed
	AG 5	RhCaES-62	brown wilted stem bases/leaves	83 n.s.	11.0 abc	7.6 bc	-
	AG BI	RhCaGo-39	brown wilted stem bases/leaves	85 n.s.	11.2 abc	8.2 bc	-
	AG-E	RhCaWS-46	crown rot	85 n.s.	10.0 c	7.6 bc	-
2	Untreated	-	-	90 ab	14.0 ab	7.9 c	-
	AG 3	RhPoWS-32	black scurf	92 a	13.3 b	7.7 c	-
	AG 2–1	RhOsSS-66	damping-off	80 ab	13.3 b	8.6 c	-
	AG 2–1	RhCfWS-83	damping-off	75 b	13.6 ab	22.1 b	-
	AG 4-HGII	RhSbSS-17	damping-off	0 c	0.0 c	100.0 a	-

^1^ n.s.: not significant. ^2^ Values followed with the same letter are not significantly different from each other (*p* = 0.05).

**Table 4:** Pathogenicity towards carrot seedlings (cv. Nantes) of six *R. solani* isolates belonging to AG 3 and AG 5, isolated from carrot and potato. Isolate BK004-1-1 (AG 4-HGII) was used as a positive control, inducing damping-off [17].

Trial No.	Anastomosis Group	Isolate No.	Symptom on Original Host Plant	Emerged Plants (%) ^1,2^	Plant Height (cm) ^2,3^	Disease Severity Index (0–100) ^2,3^
1	Untreated	-	-	83 n.s.	n.d.	0.0 c
	AG 4-HGII	BK004-1-1	damping-off	72 n.s.	n.d.	96.2 a
	AG 3	RhCaES-19	greyish-white felt-like mycelium	72 n.s.	n.d.	15.4 b
	AG 3	RhCaES-22	greyish-white felt-like mycelium	83 n.s.	n.d.	18.3 b
	AG 3	RhPoWS-30	black scurf	67 n.s.	n.d.	10.4 b
	AG 3	RhPoWS-32	black scurf	89 n.s.	n.d.	9.4 b
	AG 3	RhPoHa-49	black scurf	78 n.s.	n.d	10.7 b
	AG 5	RhCaES-38	black scurf	78 n.s.	n.d.	14.3 b
2	Untreated	-	-	78 a	3.0 ± 0.3 a	n.d.
	AG 4-HGII	BK004-1-1	damping-off	0 b	0.0 ± 0.0 d	n.d.
	AG 3	RhCaES-19	greyish-white felt-like mycelium	63 a	2.2 ± 0.3 abc	n.d.
	AG 3	RhCaES-22	greyish-white felt-like mycelium	70 a	1.5 ± 0.2 c	n.d.
	AG 3	RhPoWS-30	black scurf	68 a	2.3 ± 0.3 abc	n.d.
	AG 3	RhPoWS-32	black scurf	70 a	1.9 ± 0.2 bc	n.d.
	AG 3	RhPoHa-49	black scurf	65 a	2.0 ± 0.3 bc	n.d
	AG 5	RhCaES-38	black scurf	73 a	2.4 ± 0.3 ab	n.d.

^1^ n.s.: not significant. ^2^ Values followed with the same letter are not significantly different from each other (*p* = 0.05).^3^ n.d.: not determined.

## Data Availability

All relevant data are within the manuscript. Sequence data have been uploaded on Genbank with accession numbers MW999148–MW999205.
